# Sequencing of the complete chloroplast genome of *Rubia yunnanensis* Diels and its phylogenetic analysis

**DOI:** 10.1080/23802359.2022.2086496

**Published:** 2022-06-20

**Authors:** Wei Wang, Tao Xu, Xiangwen Song, Cunwu Chen, Dong Liu, Bangxing Han, Shanyong Yi

**Affiliations:** a Department of Biological and Pharmaceutical Engineering, West Anhui University, Luʼan, P.R. China; b Anhui Engineering Laboratory for Conservation and Sustainable Utilization of Traditional Chinese Medicine Resources, West Anhui University, Luʼan, P.R. China

**Keywords:** *Rubia yunnanensis*, complete chloroplast genome, phylogenetic analysis

## Abstract

*Rubia yunnanensis* Diels, an important medicinal herb, is mainly distributed in Yunnan province, Southwest China. In this study, the complete chloroplast genome of *R. yunnanensis* was successfully sequenced. The assembled chloroplast genome was 155,108 bp in length with an overall GC content of 36.98%, including a pair of inverted repeat (IR) regions (26,573 bp, each), respectively, a large single-copy (LSC) region (84,848 bp) and a small single-copy (SSC) region (17,114 bp). The genome contained 131 genes, comprising 85 protein-coding genes, 37 tRNA genes, eight rRNA genes, and one pseudogene. The phylogenetic analysis indicated that *R. yunnanensis* was closely related to *R. cordifolia.*

*Rubia yunnanensis* Diels (1912) is a perennial herbaceous medicinal plant of the *Rubia* genus of Rubiaceae and widely distributed in Yunnan province in Southwest China (Lan 1975). Its dried roots and rhizomes named ‘Xiaohongshen,’ is a traditional Chinese medicine for treating vertigo, insomnia, rheumatism, tuberculosis, menstrual disorders, and contusions (Yi et al. 2020). This species is used as a local alternative for *R. cordifolia* listed in Chinese Pharmacopeia. Its active ingredients include quinones, rubiaceae-type cyclopeptides and terpenoids (Fan et al. 2011). However, the phylogenetic position is unclear causing on the lack of genomic information. In this study, we reported the complete chloroplast genome of *R. yunnanensis* collected in China, which will provide the information for further bioinformatics studies of *R. yunnanensis* and the related species.

The fresh leaves of *R. yunnanensis* were collected from the medicinal botanical garden of West Anhui University, Lu’an, Anhui, China (31°77′ N, 115°93′ E). Specimens were deposited in the Herbarium of West Anhui University (voucher number WAU-XHS-20220201-1, Wei Wang, weiwangwestau@163.com). *Rubia yunnanensis* is not a protected plant in China, and the experimental material was not collected from a private or protected area that required permission. Total genomic DNA from leaves was extracted in line with a modified CTAB protocol (Doyle and Doyle 1987). The DNA was stored at −80 °C in our lab. The whole genome sequencing was performed by Hefei Biodata Biotechnologies Inc. (Hefei, China) using the Illumina Hiseq platform. The program fastp (Chen et al. 2018) and SPAdes assembler 3.10.0 (Bankevich et al. 2012) were used to filter and assemble the sequences, respectively. Then, the annotation was conducted by the GeSeq (Tillich et al. 2017) and BLASTx (Gish and States 1993) searches.

The complete chloroplast genome of *R. yunnanensis* (GenBank accession: OL467345) had a 155,108 bp in length, which contained a pair of inverted repeat (IR) regions of 26,573 bp, a large single-copy (LSC) region of 84,848 bp and a small single-copy (SSC) region of 17,114 bp. The genome contained a total of 131 genes, comprising 85 protein-coding genes, 37 tRNA genes, eight rRNA genes, and one pseudogene. Seven protein-coding, eight tRNA, and four rRNA genes were duplicated in IR regions. Among the annotated genes, nineteen genes had two exons and four genes (*paf*I, *clp*P1, and two *rps*12) had three exons. The overall GC content of *R. yunnanensis* chloroplast genome was 36.98% and those in LSC, SSC, and IR regions were 34.50%, 30.96%, and 43.87%, respectively.

To investigate the taxonomic status of *R. yunnanensis*, alignment was carried out with 39 reported chloroplast genome (full DNA) sequences of Rubiaceae (*Neolamarckia cadamba* and *Uncaria rhynchophylla* were used as outgroup taxa) using MAFFT v7.307 (Katoh and Standley 2013). The FastTree version 2.1.10 (Price 2010) was employed to produce a maximum likelihood (ML) tree. As expected, *R. yunnanensis* is mostly related to *R. cordifolia*, a species of same genus, with bootstrap support values of 100% (Figure 1). The complete chloroplast genome sequence of *R. yunnanensis* will lay a vital foundation for the conservation genetics of this species as well as for the phylogenetic studies of Rubiaceae.

**Figure 1.**
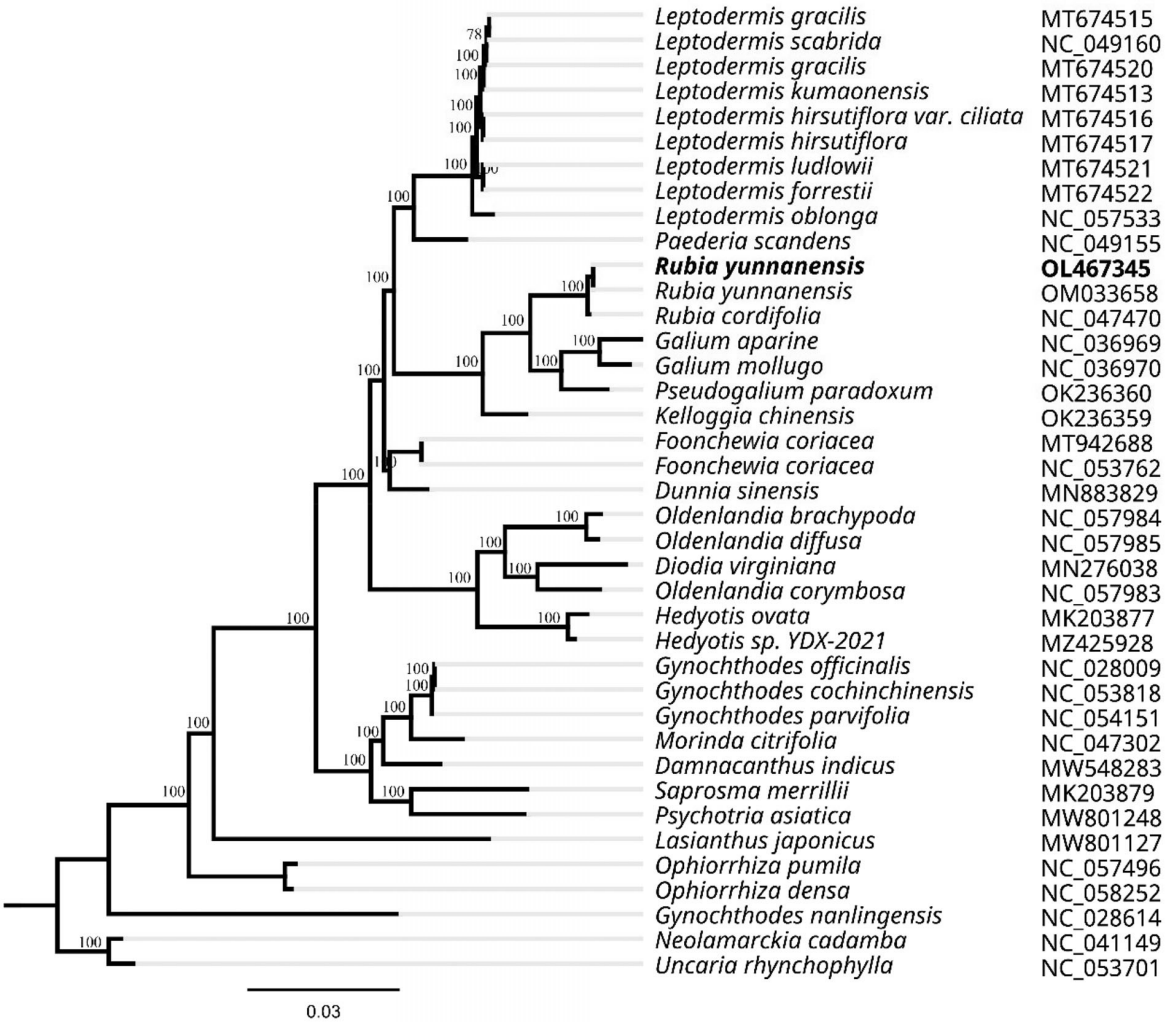
Phylogenetic tree of Rubiaceae inferred by Maximum Likelihood (ML) method based on 39 representative species. *Neolamarckia cadamba* and *Uncaria rhynchophylla* were used as outgroup taxa. A total of 1000 bootstrap replicates were computed and the bootstrap support values are shown at the branches. GenBank accession numbers were shown in Figure 1.

## Authors’ contributions

Conception and design: Yi S and Han B; data analysis and interpretation: Wang W, Xu T, and Song X; manuscript writing and revising: Wang W, Yi S, Chen C, and Liu D; All authors have read and approved the final manuscript and agree to be accountable for all aspects of the work.

## Data Availability

The genome sequence data of *R. yunnanensis* that support the findings of this study are openly available in GenBank of NCBI at (https://www.ncbi.nlm.nih.gov/) under the accession no. OL467345. The associated BioProject, SRA, and Bio-Sample numbers are PRJNA803821, SRR17898400, and SAMN25688705, respectively.
